# Acromegaly Discovered After Head Trauma: Multivessel Coronary Disease and Postoperative Meningitis Complicating a Pituitary Macroadenoma

**DOI:** 10.7759/cureus.113532

**Published:** 2026-07-28

**Authors:** Sean Rockwell, Jaxon S Boley, Nicholas Pulido, Mohammad Safa, Lila S Chertman

**Affiliations:** 1 Orthopaedic Surgery, Ross University School of Medicine, Bridgetown, BRB; 2 Orthopaedic Surgery, American University of the Caribbean, Cupecoy, SXM; 3 Medicine, Ross University School of Medicine, Bridgetown, BRB; 4 Medicine, American University of the Caribbean, Cupecoy, SXM; 5 Endocrinology, Ross University School of Medicine, Bridgetown, BRB

**Keywords:** acromegaly, coronary artery disease, growth hormone, pituitary macroadenoma, postoperative meningitis, transsphenoidal surgery

## Abstract

Acromegaly is a rare endocrine disorder caused by chronic growth hormone (GH) excess, most often from a pituitary somatotroph adenoma. Because the somatic and metabolic features develop slowly, the diagnosis is often delayed for years. We report the case of a 59-year-old man whose acromegaly was identified incidentally after a 1.6 cm pituitary macroadenoma was found on MRI obtained for trauma evaluation following a bicycle accident. History and examination revealed several long-standing features of acromegaly, including widely spaced teeth, mild prognathism, increased shoe and hand size, severe obstructive sleep apnea (OSA), worsening hypertension, recently diagnosed type 2 diabetes mellitus, and secondary hypogonadism. Biochemical testing confirmed the diagnosis with a peak insulin-like growth factor 1 (IGF-1) of 752 ng/mL and a random GH of 10.9 ng/mL. Routine preoperative cardiac evaluation identified previously unrecognized multivessel coronary artery disease requiring four coronary stents, and the resulting need for dual antiplatelet therapy delayed transsphenoidal resection by approximately seven months. Endoscopic transsphenoidal resection was performed in March 2025, with rapid GH normalization and no residual tumor on postoperative imaging. The early postoperative course was complicated by bacterial sinusitis and subsequent bacterial meningitis treated with intravenous cefepime and linezolid. By three months postoperatively, IGF-1 had normalized, and the patient had achieved substantial weight loss and improved glycemic control, although follow-up polysomnography showed worsening OSA despite biochemical remission. This case illustrates the diagnostic delay typical of acromegaly, the impact of unrecognized cardiovascular comorbidity on surgical timing, and the infectious complications that can follow transsphenoidal surgery.

## Introduction

Acromegaly is a rare endocrine disorder caused by chronic growth hormone (GH) hypersecretion, in more than 95% of cases from a benign somatotroph pituitary adenoma [1]. Recent global meta-analyses estimate a pooled prevalence of approximately 5.9 cases per 100,000 and an incidence of 0.38 cases per 100,000 person-years, with population-based studies suggesting that the true prevalence is higher as case ascertainment improves [2]. The downstream effector of GH, insulin-like growth factor 1 (IGF-1), drives most of the somatic effects of the disease, including acral and soft tissue overgrowth, facial coarsening, hypertension, insulin resistance, and accelerated cardiovascular disease [1].

The diagnosis of acromegaly is frequently delayed. Individual features such as hypertension, type 2 diabetes mellitus, and obstructive sleep apnea (OSA) are common in the general population, and the somatic changes evolve slowly. Recognition therefore usually depends on combining several features rather than identifying any one in isolation [3]. Median diagnostic delay is approximately five years, and longer delays are associated with a higher hormonal burden and more comorbidities at diagnosis [4]. Some patients are first identified incidentally when imaging obtained for an unrelated reason finds a pituitary lesion [5].

Once acromegaly is biochemically confirmed and a tumor source is identified, endoscopic transsphenoidal resection is the first-line treatment and offers biochemical remission rates of approximately 70 to 80% in suitable candidates [6,7]. Postoperative complications such as cerebrospinal fluid (CSF) leak and bacterial meningitis remain important risks, particularly when sinonasal infection or other patient-specific factors are present [8,9]. Cardiovascular complications, including hypertension, cardiomyopathy, and coronary artery disease, are also common in active acromegaly and contribute substantially to long-term mortality [10].

We describe a 59-year-old man whose acromegaly was identified incidentally after a pituitary macroadenoma was found on trauma imaging. His subsequent care was shaped by previously unrecognized multivessel coronary artery disease, which delayed definitive surgical management, and by a series of postoperative infections including influenza, bacterial sinusitis, and meningitis. The combination in a single patient - incidental discovery on trauma imaging, occult multivessel coronary artery disease requiring surgical deferral, and a postoperative course marked by influenza, bacterial sinusitis, and Haemophilus influenzae type B and Staphylococcus aureus meningitis - is not previously reported to our knowledge and provides an unusually complete illustration of the challenges that can accompany acromegaly care from diagnosis through recovery. The case illustrates several recurring themes in acromegaly care, including the long diagnostic delay, the burden of cardiometabolic comorbidity, the importance of multidisciplinary preoperative planning, and the potential for meaningful recovery even after a difficult postoperative course.

## Case presentation

The following sections describe the patient's initial clinical presentation, endocrine evaluation, imaging findings, planned treatment and reasons for delay, surgical resection, postoperative and surveillance imaging, postoperative complications, and follow-up outcomes.

Initial presentation

A 59-year-old man was referred to endocrinology in May 2024 for the evaluation of a pituitary mass and secondary hypogonadism. Four months earlier, in January 2024, he had been involved in a bicycle accident and sustained parietal-occipital skull fractures. Head imaging at the time of the accident incidentally noted enlargement of the pituitary gland, and a dedicated pituitary MRI in April 2024 demonstrated a 1.6 cm sellar macroadenoma. He was then referred for both neurosurgical and endocrine evaluation.

The history obtained at his initial endocrinology visit was notable for several features suggestive of acromegaly. Over the prior year, he had gained roughly 17 kg without any change in his diet or activity. His shoe size had increased from 10 to 11, and his hands had grown noticeably larger such that he could no longer fit the wedding ring he had worn ten years earlier. He also reported low libido and erectile dysfunction but denied nipple discharge or visual changes. His past medical history was significant for severe OSA on continuous positive airway pressure (CPAP) for four years, hypertension that had been worsening over the prior five years and now required three antihypertensive medications, and recently diagnosed type 2 diabetes mellitus (initial HbA1c 6.6% in April 2024) treated with metformin. He also had hyperlipidemia and benign prostatic hyperplasia. He was a never-smoker with social alcohol use and no personal history of cardiac disease. Family history was notable for a father with a myocardial infarction.

**Figure 1:**
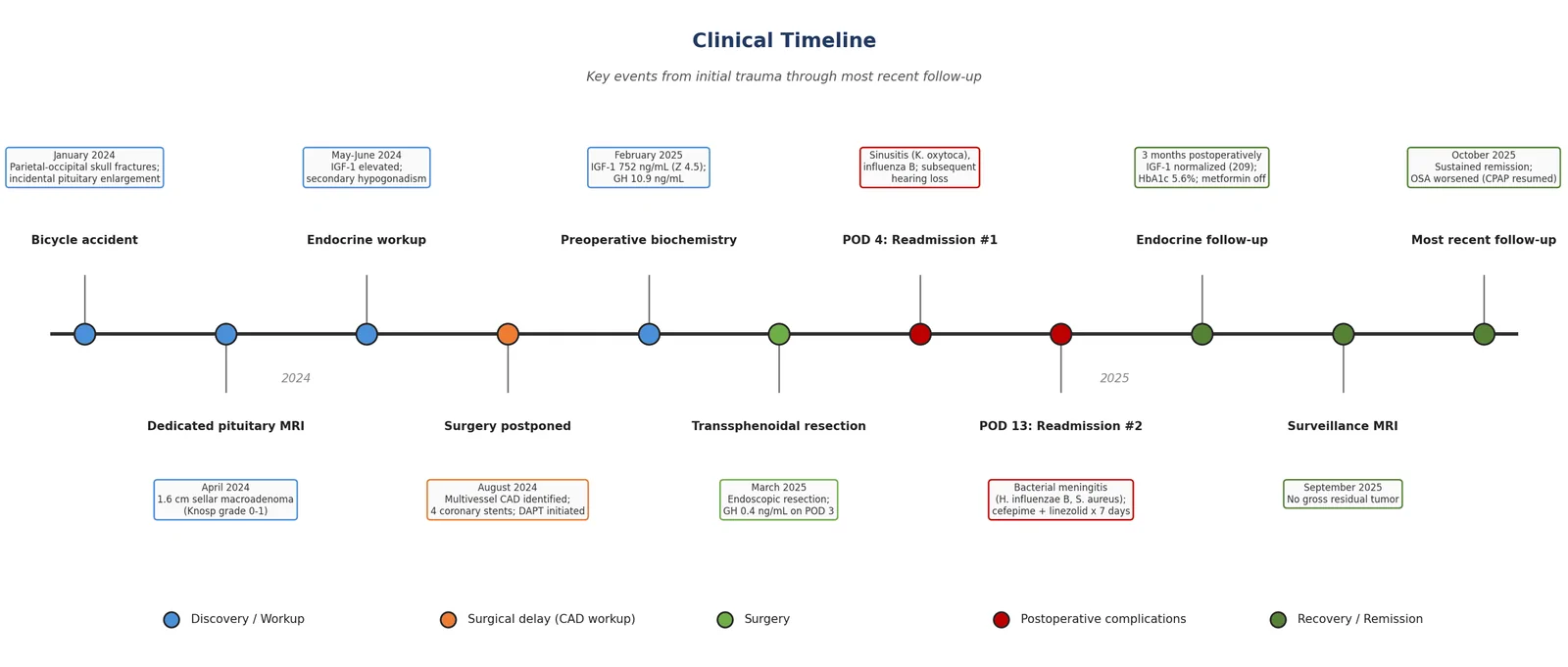
Clinical Timeline of Key Events From Initial Trauma Through Most Recent Follow-Up Graphical clinical timeline showing the sequence of events in the patient's care, from the initial bicycle accident in January 2024 through the most recent follow-up in October 2025. Events are color-coded by phase: discovery and workup (blue), surgical delay for cardiac workup (orange), transsphenoidal surgery (light green), postoperative complications (red), and recovery/remission (dark green). CAD, coronary artery disease; CPAP, continuous positive airway pressure; DAPT, dual antiplatelet therapy; GH, growth hormone; IGF-1, insulin-like growth factor 1; OSA, obstructive sleep apnea; POD, postoperative day. Figure created using Matplotlib (Python data visualization library)

On physical examination, he was 5'10" tall and weighed 203 lbs (BMI 29.1 kg/m²) with a blood pressure of 134/98 mmHg. He had widely spaced teeth, a prominent nose, and mild prognathism. He was not Cushingoid. Visual fields were grossly intact, and the remainder of the cardiopulmonary, abdominal, and neurologic examinations was unremarkable apart from a soft systolic murmur. Based on the incidental pituitary mass and the patient's clinical features, acromegaly was suspected, and a complete pituitary biochemical workup was initiated. The clinical timeline from initial trauma through most recent follow-up is summarized in Figure 1.

Endocrine evaluation

The initial pituitary panel demonstrated an elevated IGF-1 of 523 ng/mL in May 2024, rising to 648 ng/mL in June 2024 (Z-score 4.0; upper limit of normal 317 ng/mL). A repeat measurement obtained before surgery in February 2025 showed a peak IGF-1 of 752 ng/mL (Z-score 4.5) and a corresponding random GH of 10.9 ng/mL. These values, together with his clinical features, confirmed the diagnosis of acromegaly.

The remaining anterior pituitary axes were notable only for an isolated secondary hypogonadism. Total testosterone was 213 ng/dL in April 2024 with an inappropriately low luteinizing hormone of 1.4 IU/L and a normal follicle-stimulating hormone of 3.9 IU/L. Prolactin was 6.4 ng/mL, within normal limits, making a co-secreting prolactinoma unlikely. The thyroid axis (TSH 1.54 µIU/mL, free T4 1.2 ng/dL) and the hypothalamic-pituitary-adrenal axis (8 a.m. cortisol 9.5 µg/dL, ACTH 27 pg/mL, late-night salivary cortisol 0.05 µg/dL) were within normal limits, with no biochemical evidence of secondary adrenal insufficiency or hypercortisolism. Calcium (8.9 mg/dL) and parathyroid hormone (43 pg/mL) were also normal, making multiple endocrine neoplasia type 1 unlikely. Visual field testing performed at the Bascom Palmer Eye Institute in June 2024 was normal. Serial laboratory data are summarized in Table 1.

**Table 1:** Serial Endocrine and Metabolic Laboratory Data From Initial Evaluation Through Follow-up H, value above the reference range; L, value below the reference range; POD, postoperative day; ACTH, adrenocorticotropic hormone; FSH, follicle-stimulating hormone; GH, growth hormone; HbA1c, hemoglobin A1c; IGF-1, insulin-like growth factor 1; LH, luteinizing hormone; MEN1, multiple endocrine neoplasia type 1; PTH, parathyroid hormone; T4, thyroxine; TSH, thyroid-stimulating hormone; ULN, upper limit of normal. Reference cutoffs are provided in column headers where applicable; the IGF-1 upper limit of normal was 317 ng/mL for the assay used and is age-adjusted. Dashes (—) indicate measurements not obtained at that time point.

Timepoint	IGF-1 (ng/mL); ULN 317	GH (ng/mL); random <1	Total testosterone (ng/dL); 250-1100	HbA1c (%); normal <5.7	Other relevant findings/context
April 2024	—	—	213 (L)	6.6 (H)	LH 1.4 IU/L (L) (ref: 1.7–8.6); FSH 3.9 IU/L (ref: 1.5–12.4); prolactin 6.4 ng/mL (ref: 4.0–15.2); TSH 1.54 µIU/mL (ref: 0.4–4.0)
May 2024	523 (H), Z 3.4	—	—	—	First IGF-1 measurement
June 2024	648 (H), Z 4.0	—	207 (L)	—	ACTH 27 pg/mL (ref: 7.2–63); AM cortisol 9.5 µg/dL (ref: 6–23); midnight salivary cortisol 0.05 µg/dL (normal) (ref: <0.09); PTH 43 pg/mL (ref: 15–65); calcium 8.9 mg/dL (ref: 8.5–10.2) (no MEN1)
November 2024	—	—	—	5.8 (H)	On metformin
February 2025 (preoperative)	752 (H), Z 4.5	10.9 (H)	236 (L)	6.1 (H)	ACTH 38 pg/mL (ref: 7.2–63); cortisol 15 µg/dL (ref: 6–23)
POD 3	—	0.4 (normal)	—	—	Early postoperative GH normalization
POD 13 (meningitis admission)	—	—	—	—	Cortisol 14 µg/dL (ref: 6–23); ACTH 9.3 pg/mL (ref: 7.2–63); free T4 1.56 ng/dL (ref: 0.9–1.7)
Three months postoperatively	209 (normal)	—	264	5.6	Biochemical remission; testosterone normalizing; metformin discontinued

Imaging

**Figure 2:**
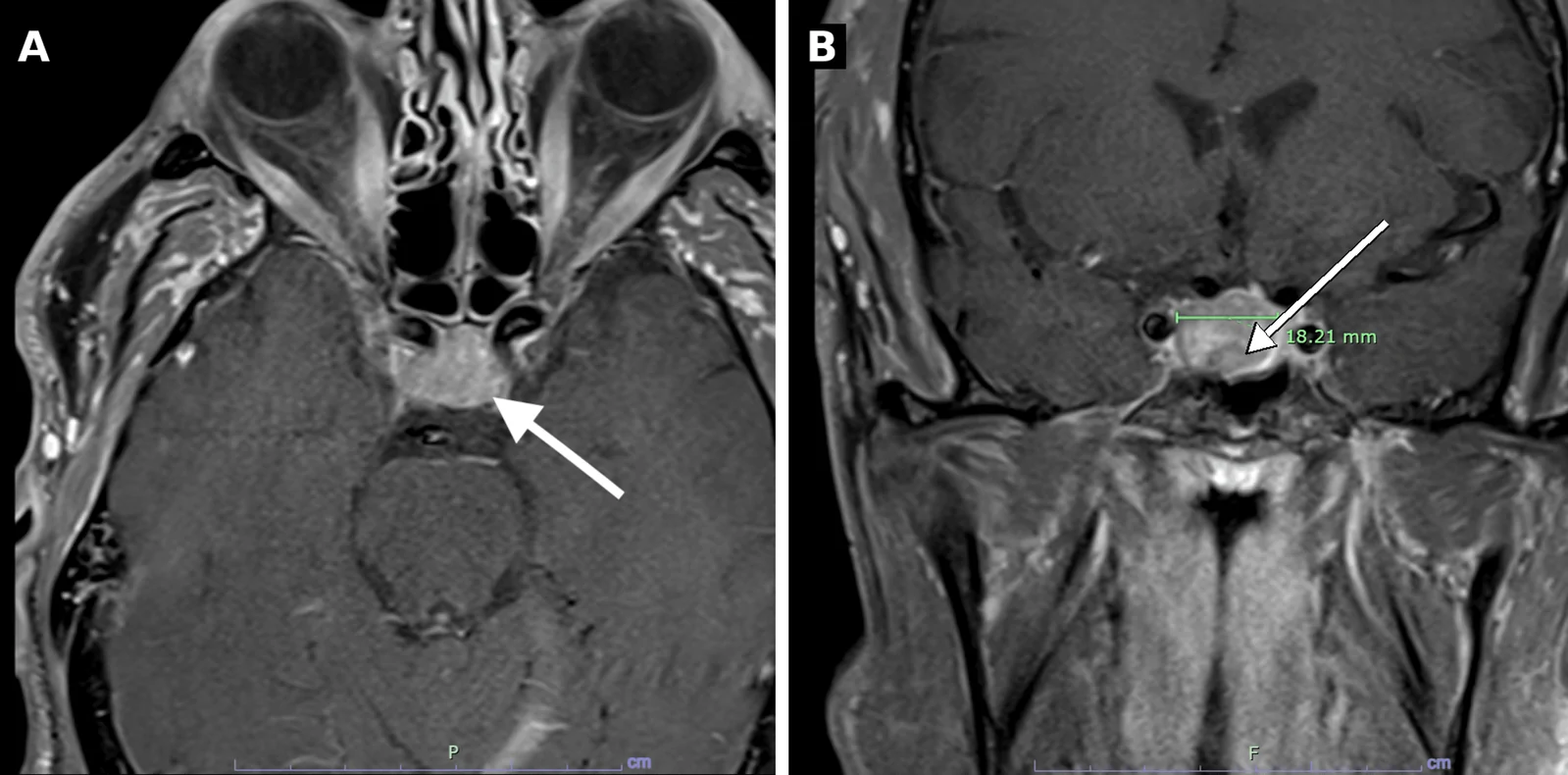
Baseline Preoperative Pituitary MRI Demonstrating Sellar Macroadenoma Initial preoperative pituitary MRI obtained in April 2024. (A) Axial T1-weighted post-contrast image with fat saturation demonstrating a heterogeneously enhancing sellar macroadenoma (arrow). (B) Coronal T1-weighted post-contrast image with fat saturation showing the lesion (arrow) with a transverse diameter of approximately 1.8 cm; residual normal pituitary tissue is compressed and displaced to the left.

The initial pituitary MRI in April 2024 demonstrated a 1.6 × 1.8 × 1.6 cm (anteroposterior × transverse × craniocaudal) sellar mass that was T1 isointense and predominantly T2 hypointense with heterogeneous enhancement (Figure 2).

There was sellar enlargement and mild effacement of the suprasellar cistern. The right lateral margin of the lesion extended to the intercarotid line (Knosp grade 1), and the left lateral margin abutted the medial tangent (Knosp grade 0). Residual normal pituitary parenchyma was compressed along the left aspect of the sella, with leftward deviation of the pituitary infundibulum. There was no mass effect on the optic chiasm. Additional findings included healing left occipital and biparietal calvarial fractures and small foci of encephalomalacia at the bilateral gyrus rectus, both consistent with the prior head trauma, as well as large bilateral mastoid effusions.

**Figure 3:**
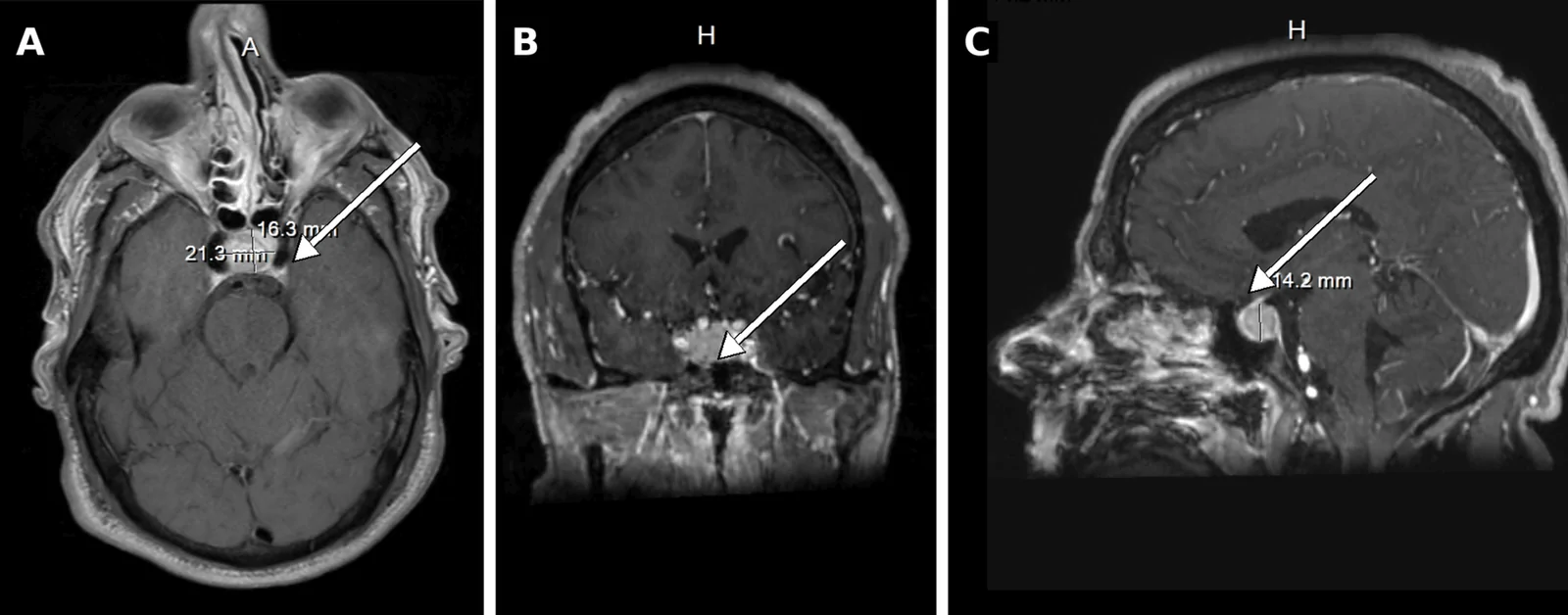
Preoperative Pituitary MRI Immediately Before Surgical Resection, Demonstrating Interval Tumor Growth Preoperative pituitary MRI obtained immediately before surgical resection in March 2025, demonstrating modest interval growth of the macroadenoma over the seven-month delay for cardiac workup. (A) Axial T1-weighted post-contrast image with fat saturation showing the lesion (arrow) with a maximum transverse dimension of 2.1 cm and anteroposterior dimension of 1.6 cm. (B) Coronal T1-weighted post-contrast image with fat saturation showing the enhancing sellar mass (arrow). (C) Sagittal T1-weighted post-contrast image with fat saturation showing the lesion (arrow) with a craniocaudal dimension of 1.4 cm.

A repeat pituitary MRI performed immediately before surgery in March 2025 demonstrated modest interval growth of the macroadenoma over the intervening seven-month delay, now measuring approximately 2.1 cm in maximum transverse dimension (Figure 3).

The lesion remained non-invasive of the cavernous sinuses and there was no mass effect on the optic chiasm.

Planned treatment and delay

After biochemical testing supported a diagnosis of acromegaly and imaging identified a pituitary macroadenoma, definitive treatment with endoscopic transsphenoidal resection was recommended. Surgery was initially scheduled for August 2024. During the routine preoperative cardiac evaluation, however, the patient was found to have previously unrecognized multivessel coronary artery disease, despite having denied chest pain or palpitations and having no known cardiac history. He underwent percutaneous coronary intervention with placement of four coronary stents. Because of the required course of dual antiplatelet therapy and the need for further cardiovascular optimization, transsphenoidal surgery was deferred by approximately seven months.

Surgery

After clearance from cardiology, the patient underwent endoscopic transsphenoidal resection of the pituitary adenoma in March 2025 at the University of Miami. He was discharged on hydrocortisone 20 mg in the morning and 10 mg in the evening, which was discontinued shortly thereafter once subsequent cortisol testing confirmed preserved adrenal function. Early postoperative laboratory studies demonstrated a rapid decline in GH into the normal range (GH 0.4 ng/mL on postoperative day (POD) 3). Surgical pathology confirmed pituitary adenoma, with the principal specimen measuring approximately 2.3 × 1.7 cm and an additional smaller fragment of 0.3 × 0.2 × 0.2 cm. The slight difference between the imaging and pathology measurements likely reflects the ex vivo state of the resected specimen and the loss of tissue compression once removed from the sella.

Postoperative and surveillance imaging

**Figure 4:**
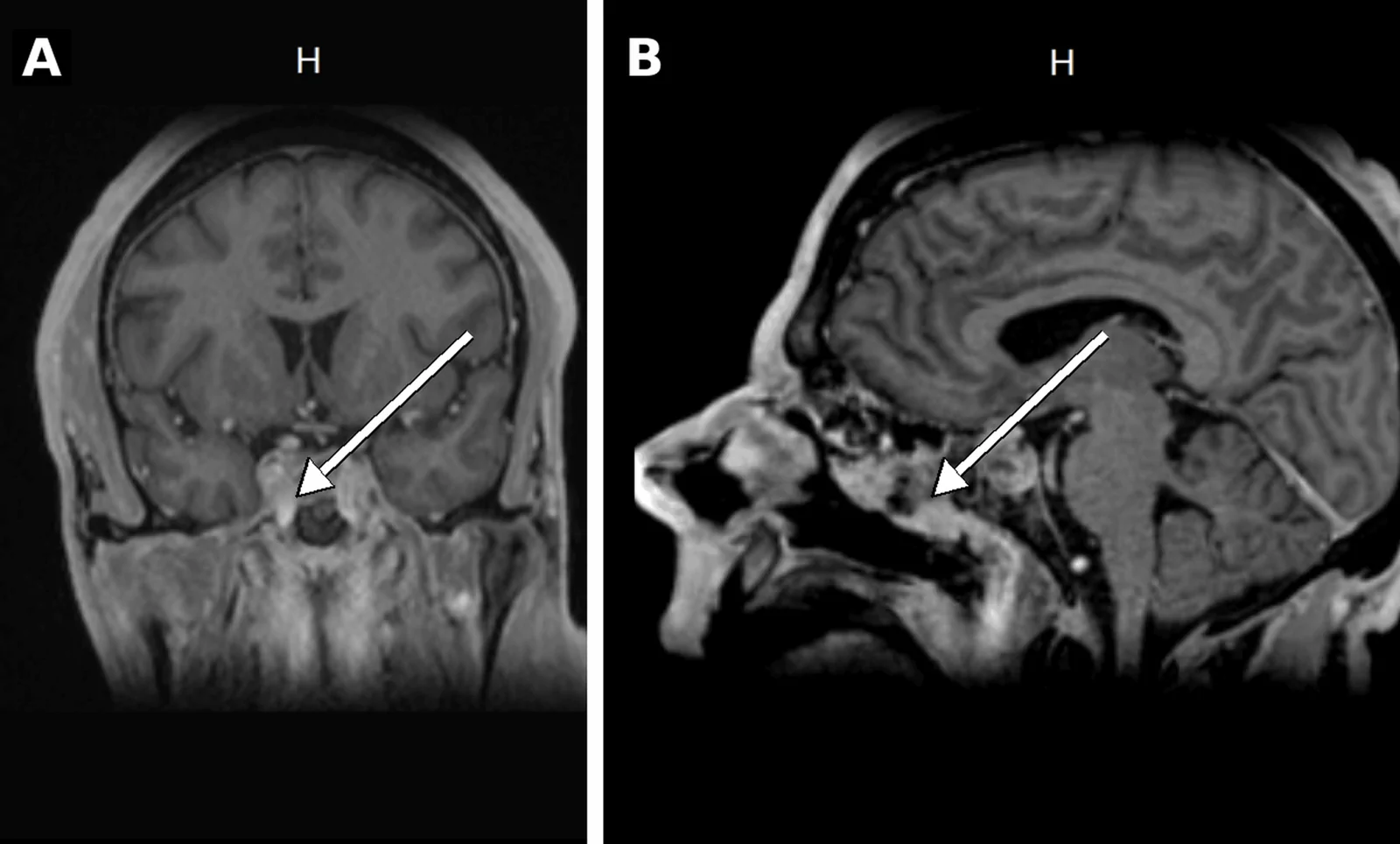
Immediate Postoperative Pituitary MRI on Postoperative Day 1 Showing Complete Tumor Resection Immediate postoperative pituitary MRI obtained on postoperative day 1, demonstrating expected postsurgical changes without evidence of residual tumor. (A) Coronal T1-weighted post-contrast image showing the surgical cavity (arrow) with postoperative material and no residual enhancing tumor. (B) Sagittal T1-weighted post-contrast image showing the postoperative sellar changes (arrow).

The immediate postoperative MRI obtained on POD 1 showed expected postsurgical changes within the central and right aspects of the sella with hemorrhage and postoperative material in the surgical cavity (Figure 4).

**Figure 5:**
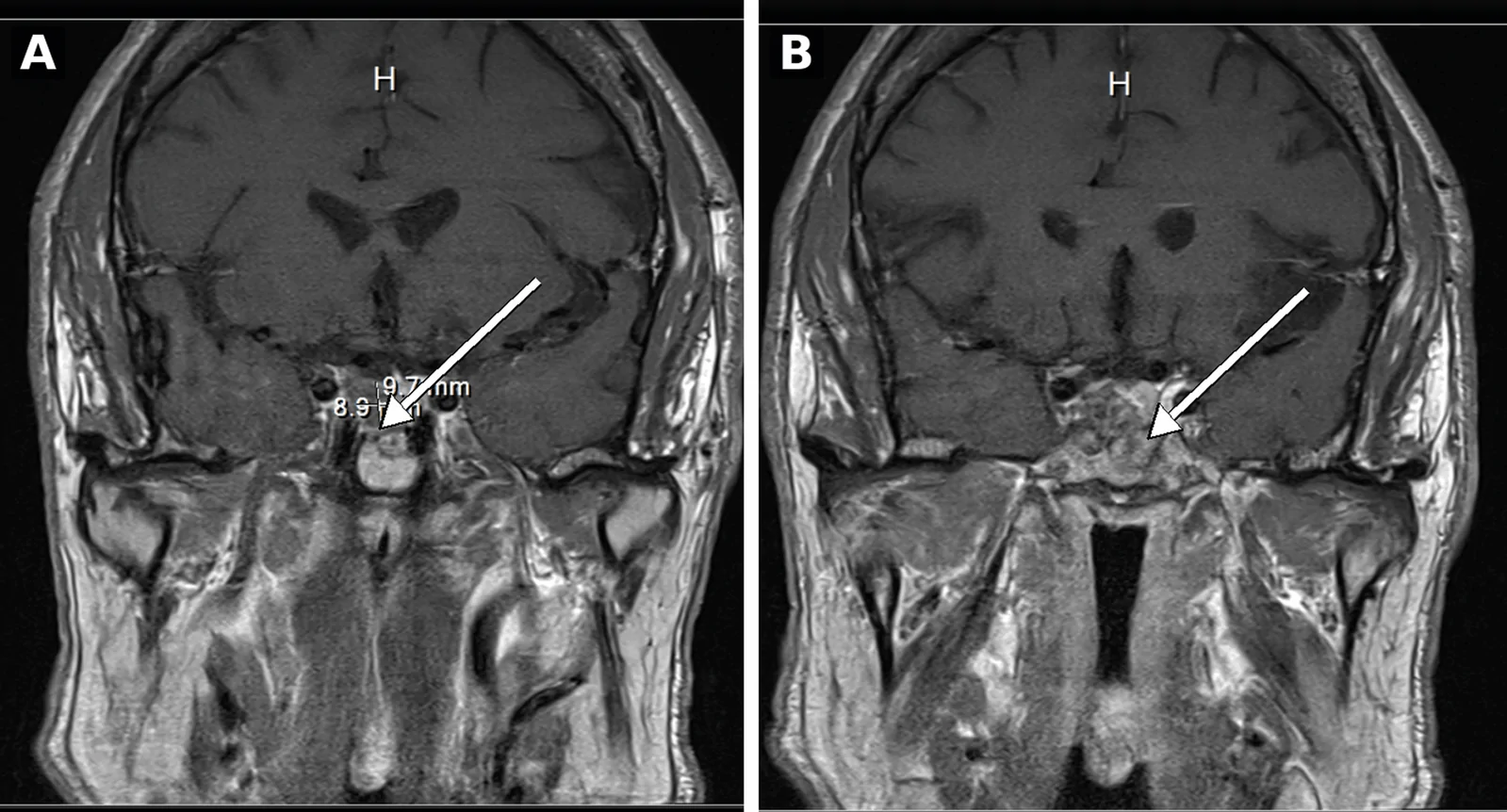
Surveillance Pituitary MRI at Six Months Confirming Durable Resection Surveillance pituitary MRI obtained in September 2025, approximately six months after surgery, confirming durable resection of the macroadenoma. (A) Coronal T1-weighted post-contrast image showing the residual surgical cavity (arrow) measuring approximately 9.7 × 8.9 mm without a gross residual tumor. (B) Coronal T1-weighted post-contrast image at a slightly different level showing no residual enhancing tumor.

Homogeneously enhancing residual normal pituitary tissue was identified along the left side of the sella. There was no evidence of residual tumor and no mass effect on the optic chiasm. A surveillance MRI in September 2025 confirmed durable interval resection of the macroadenoma without gross residual tumor, with a residual surgical cavity measuring approximately 9.7 × 8.9 mm (Figure 5).

Postoperative complications

On POD 4, the patient was readmitted with fever, headache, and respiratory symptoms. Evaluation was notable for concurrent rhinovirus and influenza B infection, mild acute hypoxic respiratory failure, and postoperative sinusitis with sinus cultures positive for Klebsiella oxytoca. He completed antimicrobial therapy with oral doxycycline and amoxicillin-clavulanate and was discharged on POD 8. He subsequently developed hearing loss; the etiology was uncertain, with potential contributors including the sinonasal infection, postoperative inflammation, and antimicrobial therapy.

Five days later, on POD 13, the patient required a second readmission for bacterial meningitis. CSF analysis demonstrated a hazy, xanthochromic appearance with marked neutrophilic pleocytosis (nucleated cell count 2,378 with 92% neutrophils), markedly low glucose (<20 mg/dL), and elevated protein (372 mg/dL). A multiplex meningitis/encephalitis PCR panel performed on the CSF was positive for both Haemophilus influenzae type B and Staphylococcus aureus. Additional microbiological data, including culture results and pathogen-versus-contaminant assessment of the two organisms, were not available for retrospective review; the diagnosis of bacterial meningitis and treatment decisions were made based on the combination of clinical presentation, CSF profile, and multiplex panel results. Of note, the patient had a documented history of methicillin-resistant Staphylococcus aureus (MRSA) buccal cheek infection approximately one year prior, suggesting possible prior staphylococcal colonization. Imaging during this admission did not demonstrate findings of meningitis or other acute intracranial process; the diagnosis was made clinically and confirmed by CSF analysis. Given the temporal relationship and the anatomic proximity of the sphenoid sinus to the surgical site, the preceding postoperative sinusitis was considered a plausible source for the meningitis. He was treated with intravenous cefepime and linezolid for seven days, with gradual clinical improvement. During this admission, imaging also identified superficial thrombophlebitis of the right basilic and cephalic veins.

Follow-up and outcome

Despite a difficult postoperative course, the patient improved steadily over the following months. He had already lost approximately 30 lbs within the first two weeks after surgery, with subsequent weight stabilization at a healthier baseline, and he reported a return to his usual energy level. Follow-up endocrine testing three months postoperatively showed normalization of IGF-1 (209 ng/mL), consistent with biochemical remission of acromegaly. Thyroid and adrenal function remained intact, and there was no clinical or biochemical evidence of diabetes insipidus. Total testosterone normalized to 264 ng/dL without the need for testosterone replacement, indicating resolution of his secondary hypogonadism after definitive treatment of the adenoma. Glycemic control also improved substantially, allowing stepwise reduction and ultimate discontinuation of metformin with an HbA1c of 5.6% at three months postoperative. This trajectory is consistent with prior reports that GH excess can drive insulin resistance and that biochemical remission of acromegaly is associated with improved glucose tolerance.

Although the patient initially reported subjective improvement in his OSA symptoms after surgery, two follow-up sleep studies, including an in-laboratory polysomnography, demonstrated objectively worse OSA compared with preoperative baseline as reported by the interpreting sleep specialist, and CPAP therapy was continued. Granular apnea-hypopnea index values from the pre- and postoperative polysomnography studies were not available for retrospective quantitative comparison. Surveillance MRI in September 2025 confirmed no gross residual tumor. At the most recent follow-up in October 2025, the patient remained clinically stable, off antidiabetic therapy, and with sustained normalization of IGF-1; continued endocrine surveillance every six months was planned.

## Discussion

Diagnostic delay remains a hallmark challenge in acromegaly, with delays of several years frequent due to gradual onset and nonspecific early features [4]. Longer delays are associated with higher GH levels at diagnosis and an increased burden of comorbidities [4]. In our patient, the diagnosis was not clinically suspected and was instead made incidentally after MRI imaging for trauma, illustrating a recognized pathway in which pituitary lesions are discovered during unrelated investigations [5]. In retrospect, the patient exhibited classic features at diagnosis, including acral enlargement, facial changes, and metabolic disturbances, which are among the most commonly reported presenting features [3].

Acromegaly is strongly associated with hypertension, type 2 diabetes mellitus, and OSA, all of which contribute substantially to long-term morbidity [11]. Diabetes in particular has been shown to increase both overall and cardiovascular mortality in acromegaly [12], and OSA is highly prevalent due to soft tissue overgrowth and upper airway obstruction [13]. The patient's history of these conditions prior to treatment reflects the cumulative burden of prolonged GH excess. Notably, his diabetes essentially resolved with biochemical remission, allowing complete discontinuation of metformin within months of surgery, which is consistent with GH-induced insulin resistance contributing to his hyperglycemia, though other factors including weight loss and lifestyle changes may also have contributed.

Current guidelines recommend measuring serum IGF-1 in patients with associated conditions including OSA, type 2 diabetes mellitus, hypertension, debilitating arthritis, and carpal tunnel syndrome, even without an overt acromegaly phenotype [7]. Our patient had been treated for severe OSA on CPAP for four years, had progressively worsening hypertension over five years requiring three medications, and had recently been diagnosed with type 2 diabetes mellitus. Despite meeting multiple recognized screening criteria, IGF-1 was not measured until imaging incidentally identified a sellar mass, illustrating the under-recognition of acromegaly in primary care and non-specialty settings [4]. Endoscopic transsphenoidal resection is the first-line treatment for acromegaly, achieving biochemical remission in approximately 70 to 80% of patients, with higher rates predicted by smaller tumor size, lower preoperative GH and IGF-1, lack of cavernous sinus invasion, and surgery at high-volume centers [6,14]. Our patient's tumor was relatively small (1.6 cm) with non-invasive cavernous sinus extension (Knosp grade 0-1), favorable factors that likely contributed to his durable biochemical remission.

A defining feature of this case was the unrecognized cardiovascular disease that delayed definitive surgical management. Cardiovascular complications are common in acromegaly and include hypertension, cardiomyopathy, valvular disease, atherosclerosis, coronary artery disease, and arrhythmias [10]. Coronary artery disease has been reported in approximately 10% of acromegaly patients at diagnosis [10]; whether this represents an excess over the general population is debated, but multiple cardiovascular risk factors typically coexist in this population. Our patient had multivessel disease requiring four coronary stents despite no reported chest pain, palpitations, or known cardiac history, illustrating that cardiovascular disease in this population can be clinically silent. The Pituitary Society consensus recommends thorough cardiovascular evaluation at diagnosis and before major surgery [15], and discovery of CAD before elective transsphenoidal surgery in our patient supports the value of thorough preoperative cardiac evaluation in patients with longstanding GH excess. The need for dual antiplatelet therapy after stent placement also created a specific challenge for surgical timing: proceeding on dual antiplatelet therapy carried unacceptable operative risk, while premature interruption in the early period after stent placement risked in-stent thrombosis. Multidisciplinary coordination between endocrinology, cardiology, and neurosurgery resulted in deferral of surgery for approximately seven months, during which the patient was medically optimized.

Transsphenoidal surgery is generally safe but carries risks of CSF leak and central nervous system infections [8]. CSF leakage is a primary risk factor for postoperative meningitis, and additional risks include diabetes mellitus, prior or concurrent sinonasal infection, and prolonged operative time [8,9]. Our patient developed bacterial sinusitis with Klebsiella oxytoca within days of discharge, followed five days later by readmission for bacterial meningitis. The temporal sequence and the anatomic proximity of the sphenoid sinus to the surgical site raise the possibility that the preceding sinonasal infection may have contributed to bacterial spread to the meninges, although direct causal attribution cannot be established. CSF was positive for Haemophilus influenzae type B and Staphylococcus aureus by PCR, an unusual organism profile for community-acquired adult meningitis but consistent with sinonasal flora introduced through a postoperative pathway. The patient's prior history of MRSA infection further supports possible staphylococcal colonization. Sensorineural hearing loss is a recognized sequela of bacterial meningitis, occurring in approximately 25 to 30% of patients across mixed adult and pediatric cohorts. The association is particularly strong with H. influenzae infection [16]. The hearing loss in our patient is consistent with this established complication, although contributions from antimicrobial exposure or postoperative inflammation cannot be excluded.

Despite these complications, the patient demonstrated substantial clinical and biochemical improvement after surgery. Reductions in IGF-1 are commonly accompanied by improvements in OSA severity, although this is not universal; OSA persists in a substantial proportion of patients despite biochemical remission, particularly when soft tissue and skeletal contributors to upper airway obstruction are not fully reversible [13]. In our patient, IGF-1 normalization was accompanied by marked improvement in glycemic control and weight loss, but follow-up polysomnography demonstrated worsening OSA, illustrating this divergence between biochemical and respiratory outcomes. Biochemical remission nonetheless remains the goal, as normalization of GH and IGF-1 has been associated with restoration of life expectancy approaching that of the general population [6].

## Conclusions

This case highlights several important lessons in the diagnosis and management of acromegaly. The diagnosis remains frequently delayed; in our patient, the disease was suspected only after trauma-related imaging, despite years of widely spaced teeth, increasing acral size, severe OSA, worsening hypertension, and newly diagnosed type 2 diabetes mellitus. Each of these features should prompt earlier consideration of acromegaly. The case also illustrates the substantial cardiovascular burden of the disease: occult multivessel coronary artery disease materially altered the surgical timeline and reinforces the value of thorough cardiovascular evaluation before transsphenoidal surgery, particularly in patients with longstanding GH excess. Endoscopic transsphenoidal resection is generally well tolerated, but postoperative infections including sinusitis and bacterial meningitis can produce meaningful morbidity and warrant prompt evaluation when fever, headache, or new neurologic symptoms develop in the early postoperative period. Despite a difficult postoperative course, our patient achieved biochemical remission with normalization of IGF-1, substantial weight loss, and improved glycemic control. Early recognition, careful preoperative risk stratification, and close postoperative follow-up by a multidisciplinary team remain the foundation of good outcomes in acromegaly.
